# Radiation-induced nanogel engineering based on pectin for pH-responsive rutin delivery for cancer treatment

**DOI:** 10.1007/s00210-024-03573-y

**Published:** 2024-11-14

**Authors:** Khaled El-Adl, Mohamed M. Ghobashy, Amel F. M. Ismail, Ahmed El-morsy, Nabil A. Shoman

**Affiliations:** 1 Chemistry Department, Faculty of Pharmacy, Heliopolis University for Sustainable Development, Cairo, Egypt; 2 Pharmaceutical Medicinal Chemistry and Drug Design Department, Faculty of Pharmacy (Boys), Al-Azhar University, Nasr City, 11884 Cairo Egypt; 3 Radiation Research of Polymer Chemistry Department, National Center for Radiation Research and Technology (NCRRT), Egyptian Atomic Energy Authority (EAEA), Nasr City, P.O. Cairo Egypt; 4 Drug Radiation Research Department, National Center for Radiation Research and Technology (NCRRT), Egyptian Atomic Energy Authority (EAEA), Cairo, Egypt; 5 Pharmaceutical Organic Chemistry Department, Faculty of Pharmacy (Boys), Al-Azhar University, Nasr City, 11884 Cairo Egypt; 6 Pharmaceutical Chemistry Department, College of Pharmacy, The Islamic University, Najaf, Iraq; 7 Department of Pharmaceutics and Pharmaceutical Technology, Faculty of Pharmacy, Ahram Canadian University, Giza, Egypt

**Keywords:** Pectin, Nanogel, Drug delivery, Gamma irradiation, Rutin

## Abstract

This research investigates the formulation of a nanogel complex using pectin and poly(acrylic acid) (PAAc) to encapsulate rutin. The nanogel’s pH-responsive behavior and its potential as a targeted drug delivery platform are investigated. The gamma irradiation–induced crosslinking mechanism is elucidated, highlighting its role in creating a stable three-dimensional network structure within the polymer matrix. Fourier transform infrared spectroscopy analysis sheds light on the molecular interactions within rutin and the nanogel-rutin complex. The pH-responsive behavior of the nanogel is explored, showcasing its ability to release rutin selectively in response to pH variations and displaying high physical and chemical stability. Transmission electron microscopy imaging provides visual insights into nanogel morphology and interactions. The cumulative drug content from the nanogel was 86.14 ± 2.61%. The pH-dependent release profile of the nanogel was examined, demonstrating selective rutin release in response to varying pH levels. Cytotoxicity studies were conducted against four human cancer cell lines—HepG2, A549, MCF-7, and HCT-116 showing significant reductions in IC_50_ values, indicating enhanced therapeutic efficacy. Additionally, molecular docking studies revealed strong binding interactions of rutin with VEGFR-2 and EGFR^T790M^. Our nanogel compound 5 significantly reduced the IC_50_ values for HepG2, A549, MCF-7, and HCT-116 cells by 58.19%, 81.29%, 71.81%, and 67.16%, respectively. Furthermore, it lowered the IC_50_ values for VEGFR-2 and EGFR^T790M^ by 29.66% and 68.18%, respectively.

**Graphical Abstract:**

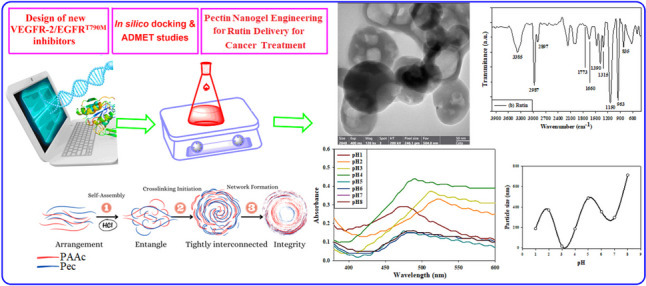

## Introduction

Cancer is a leading cause of global mortality and poor quality of life, characterized by unregulated cell proliferation and the absence of programmed cell death. In 2020, cancer incidence and mortality reached alarming levels, with an estimated 19.3 million new cases and 10 million deaths worldwide. Asia bears the highest cancer burden, accounting for 49.3% of cases and 58.3% of deaths. Europe, despite comprising only 9.7% of the world’s population, accounted for 22.8% of cases and 19.6% of deaths. The Americas also face a significant burden, representing 20.9% of global cases and 14.2% of deaths (Sobti et al. 2024). The integration of nanotechnology into drug delivery systems has led to significant progress in the field of oncotherapy (El-Adl et al. 2024). A key challenge with drug delivery systems is achieving precise targeting of tumor cells, which is crucial for avoiding potential negative effects on healthy cells from the therapeutic agents (Li et al. 2023). Nanoparticle carriers have become increasingly popular due to their potential therapeutic applications, particularly in cancer treatment, as they provide an efficient and selective means for delivering therapeutic substances to desired cells and tissues (Kenchegowda et al. 2021). In the global effort to combat cancer’s devastating impact, researchers are actively engaged in creating innovative carrier systems designed for precise targeted delivery of anticancer drugs, sparing healthy tissues from harm. Among these options, chemotherapy is a widely employed method for eliminating cancer cells. However, conventional chemotherapy delivery systems are accompanied by drawbacks such as harmful effects on healthy cells, leading to severe side effects that may even lead to fatality. Additionally, tumor cells may develop resistance to chemotherapy drugs, diminishing the treatment’s efficacy (Debela et al. 2021).

Pectin-based drug delivery systems have gained prominence in pharmaceutical applications due to their unique properties, particularly in cancer treatment. Pectin is a naturally occurring polysaccharide found in the cell walls of fruits and vegetables, known for its biodegradability, biocompatibility, and ability to form stable nanogel systems. These characteristics make pectin an excellent candidate for targeted drug delivery, as it can break down into non-toxic products within the body, minimizing long-term toxicity. Its biocompatibility ensures low immunogenicity, making it suitable for various therapeutic applications (Kedir et al. 2022). Moreover, pectin’s capacity to form nanogels enhances hydrophobic drug encapsulation and controlled release, allowing for targeted delivery to tumor sites. In parallel, the role of vascular endothelial growth factor receptor (VEGFR) and epidermal growth factor receptor (EGFR) inhibitors in cancer treatment is critical. VEGFR is essential for tumor angiogenesis forming new blood vessels that supply nutrients to tumors while EGFR is involved in cell proliferation and survival (Kapoor et al. 2024). Overexpression or mutation of EGFR is common in various cancers, making it a significant target for therapy (Karlsen et al. 2021). Dual inhibition of VEGFR and EGFR has shown therapeutic benefits by enhancing efficacy and addressing resistance mechanisms often encountered with monotherapy. This combined approach improves treatment outcomes and provides a strategic method to combat cancer’s adaptive resistance to single-agent therapies. Thus, integrating pectin-based drug delivery systems with dual VEGFR/EGFR inhibition represents a promising avenue for enhancing cancer treatment efficacy. Flavonoids, particularly rutin, have garnered attention for their potential role in cancer prevention and therapy due to their antiproliferative, antioxidant, and anti-inflammatory properties (Wang et al. 2023). Rutin, a quercetin glycoside, is known to inhibit key signaling pathways involved in tumor growth, specifically targeting vascular endothelial growth factor receptor (VEGFR) and epidermal growth factor receptor (EGFR) signaling (Nouri et al. 2020). By inhibiting VEGFR, rutin disrupts angiogenesis the formation of new blood vessels that tumors rely on for growth and metastasis thereby limiting nutrient supply to the tumor (Liskova et al. 2020). Concurrently, its action on EGFR helps to suppress cell proliferation and survival, which are critical processes in cancer progression (Wee and Wang 2017). Moreover, rutin enhances the therapeutic potential of targeted drug delivery systems by providing a dual-action mechanism against tumor growth and metastasis. This dual inhibition improves the effectiveness of cancer treatments and addresses resistance mechanisms that often limit the efficacy of single-agent therapies (Jin et al. 2023). Integrating rutin into drug delivery systems can optimize therapeutic outcomes by leveraging its natural properties to enhance bioavailability and target specificity, thereby offering a promising strategy for more effective cancer treatment (Mondal et al. 2023), complemented by explored innovative hydrogel for environmental application, demonstrating the efficacy of pH-sensitive polymer (Ghobashy et al. 2022; Abd El-Sattar et al. 2022).

Within this context, polymeric carriers like micelles (Kaur et al. 2022; Figueiras et al. 2022) and hydrogels (Thang et al. 2023; Xie et al. 2021) have been harnessed for the delivery of therapeutic proteins and medications. Nanogels, a subset of particulate DDS, have also been under investigation (Huang et al. 2018; Ranote et al. 2022). As drug carriers, nanogels exhibit advantageous features, including controllable size, a notable loading capacity, and the potential for responsiveness to environmental stimuli. Of particular importance is their amenability to surface conjugation with receptor-specific molecules, enabling targeted delivery. Lately, stimuli-sensitive nanogels have attracted considerable interest as a carrier for drugs due to their ability to exhibit swelling and deswelling in response to variations in ionic strength, pH, or temperature. Additionally, researchers have the opportunity to investigate a range of polymeric combinations, each varying in terms of functionalization and flexible chemistry (Kaur et al. 2022). This research aims to investigate the effect of a nanogel system designed to target rutin as an anticancer agent in HCT-116, MCF-7, A549, and HepG2 cancer cell lines.

Rutin is a flavonoid commonly found in daily consumables like fruits, vegetables, tea, and wine. It is a glycoside of quercetin and rutinose, and it possesses various pharmacological properties that are beneficial for human health. These properties include anticancer, anti-inflammatory, neuroprotective, anti-proliferative, anti-carcinogenic, and anti-oxidative stress effects (Ganeshpurkar and Saluja 2017). Rutin has also been found to be useful in treating and preventing rosacea (Tsiskarishvili et al. 2014). Its strong antioxidant activity is believed to contribute to its therapeutic effects in biological systems, particularly in cancer cells. Flavonoids like rutin are predominantly present in fruits, vegetables, grains, and other components of human diets (Liskova et al. 2021). Numerous studies have demonstrated that flavonoids, as polyphenolic compounds, exhibit antiproliferative and cytoprotective effects and can induce apoptosis in several cancer cell lines (Talib et al. 2022; Mahmud et al. 2023).

Angiogenesis, controlled by several angiogenic regulators, is a crucial feature for the growth, invasion, and spread of cancer. Of the various angiogenic regulators, vascular endothelial growth factor (VEGF) and epidermal growth factor (EGF) emerge as the most crucial factors in the regulation of tumor angiogenesis. Both are implicated in analogous downstream signaling pathways and may serve distinct functions in oncogenesis and the development of therapeutic resistance (Melegh and Oltean 2019). Therefore, a combination therapy targeting both VEGF and EGFR is a viable approach for treating EGFR-mutant non-small cell lung cancer.

Vascular endothelial growth factor receptor-2 (VEGFR-2) is pivotal in signal transduction during both pathological and physiological angiogenesis. The activation of VEGFR-2 results in the pairing of two monomeric receptors and tyrosine phosphorylation in the intracellular domain of the receptor, initiating a signal transduction pathways that activate subsequent cascades (Fan et al. 2019). These processes eventually culminate in tumor angiogenesis and trigger vascular permeability, proliferation, migration, and invasion (Alsaif et al. 2021). Notably, VEGFR-2 is frequently overexpressed or excessively activated in various cancers, including ovarian, thyroid, breast, renal, and hepatocellular carcinoma (Abdelgawad et al. 2022a). Consequently, targeting this signaling pathway has been identified as a crucial strategy for anti-angiogenic therapy and the inhibition of cancer progression (Shojaei 2012). In recent decades, the FDA has approved many potent VEGFR-2 inhibitors (Modi and Kulkarni 2019), including Sorafenib (Nexavar)® and Sunitinib.

Moreover, constitutively activating mutations or overexpression of EGFR impair the EGFR signaling system in a variety of cancer types, including hepatocellular, lung, and breast cancer (Modi and Kulkarni 2019). Additionally, it is widely known that kinase domain mutations and extracellular domain deletions cause ligand-independent activation of EGFR in lung cancer and glioblastoma (Hsu and Hung 2016). Consequently, EGFR tyrosine kinase (EGFR-TK) has become a key model in the seek to develop new anticancer therapies.

VEGFR-2 and EGFR both contribute to the progression of diverse tumor forms and are associated with pathological conditions. They exhibit a close connection and share common signaling cascades. The efficient interplay between VEGFR-2 and EGFR is firmly established: blocking VEGFR-2 signaling can enhance the effectiveness of EGFR inhibitors, while independent activation of VEGFR-2 may lead to resistance against these EGFR inhibitors (Castro Barbosa et al. 2014). Consequently, it appears that simultaneously targeting the EGFR and VEGFR signaling pathways is a promising strategy for tumor therapy (Garofalo et al. 2011).

Building upon our preceding research in designing and synthesizing new anticancer agents (El-Adl et al. 2021; El-Adl et al. 2020a; El-Adl et al. 2020b; El-Adl et al. 2020c; El-Adl et al. 2022; Sayed et al. 2021; Saleh et al. 2020; Abd El-Sattar et al. 2021; Abdelgawad et al. 2022b), we have developed a pectin/poly(acrylic acid) nanogel system containing rutin for targeted drug delivery. This nanogel serves as a dual inhibitor of EGFR-mutant and VEGFR-2 tyrosine kinases, aimed at treating non-small cell lung cancer (A549), hepatocellular carcinoma (Hep-G2), breast cancer (MCF-7), and human colorectal carcinoma (HCT-116).

**Fig. 1:**
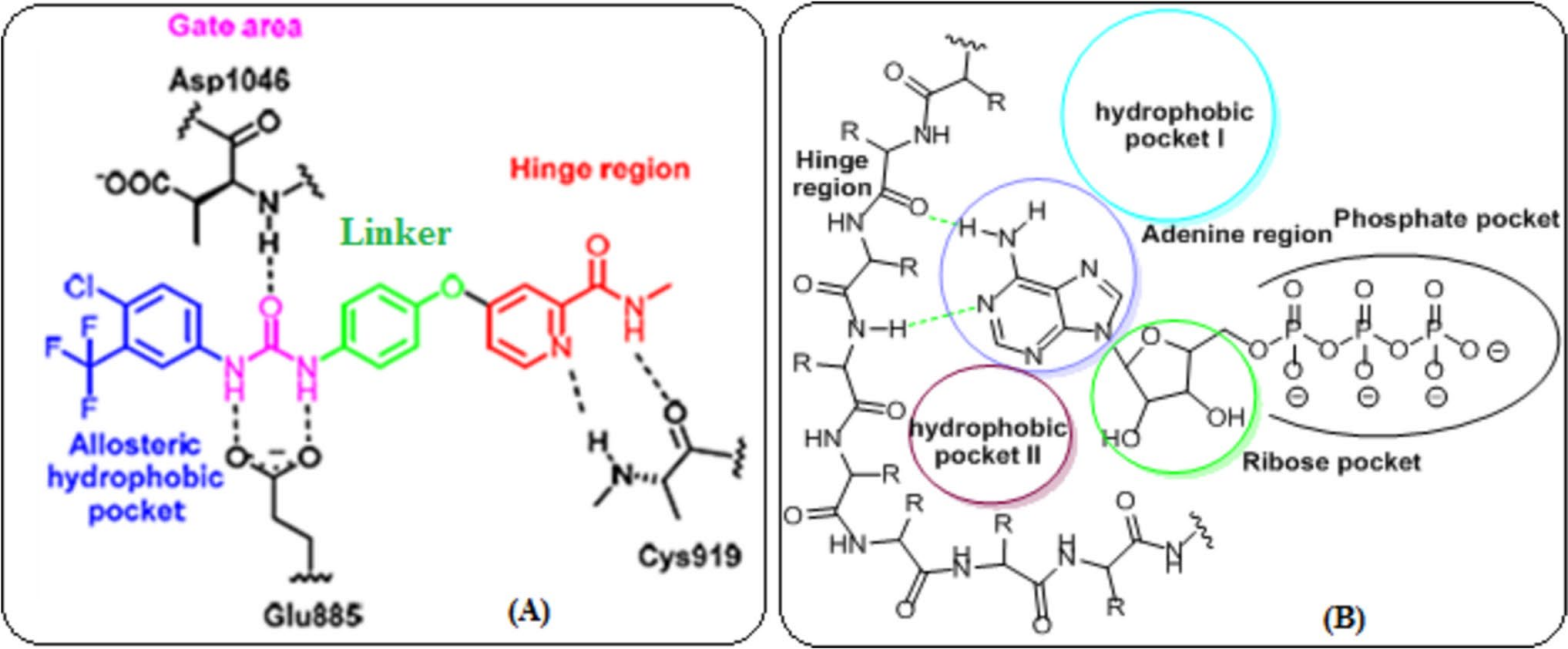
The active site of VEGFR-2 (**A**) and EGFR-TK (**B**)

VEGFR-2 inhibitors are categorized into three types. Type I inhibitors, which are ATP binding inhibitors, bind to the hinge region and establish hydrogen bonds with Cys919. After binding the ATP site, type II inhibitors penetrate the gate region and enter the nearby allosteric hydrophobic pocket. Conversely, the allosteric hydrophobic pocket is the primary target of type III inhibitors. Type II inhibitors are preferred due to their selectivity and affinity, as they effectively extend the inhibition of tyrosine kinase (TK) activity as they increase the residence time of the drug-target interaction (Abdel-Mohsen et al. 2020). Consequently, various approaches have been employed to identify new Type II VEGFR-2 inhibitors (Fig. 1A).

Regarding EGFR-TK, its ATP-binding pocket consists of five crucial regions can be targeted to enhance the pharmacokinetics of inhibitors (Fig. 1B) (Gandin et al. 2015). These regions include (a) the adenine region, where specific amino acids bond with the adenine ring by hydrogen bonds; (b) the hydrophilic ribose region; (c) hydrophobic region I, which is crucial for the selectivity of inhibitors; (d) hydrophobic region II, which can be utilized to enhance the specificity of inhibitors; and (e) the phosphate-binding sector.

**Fig. 2:**
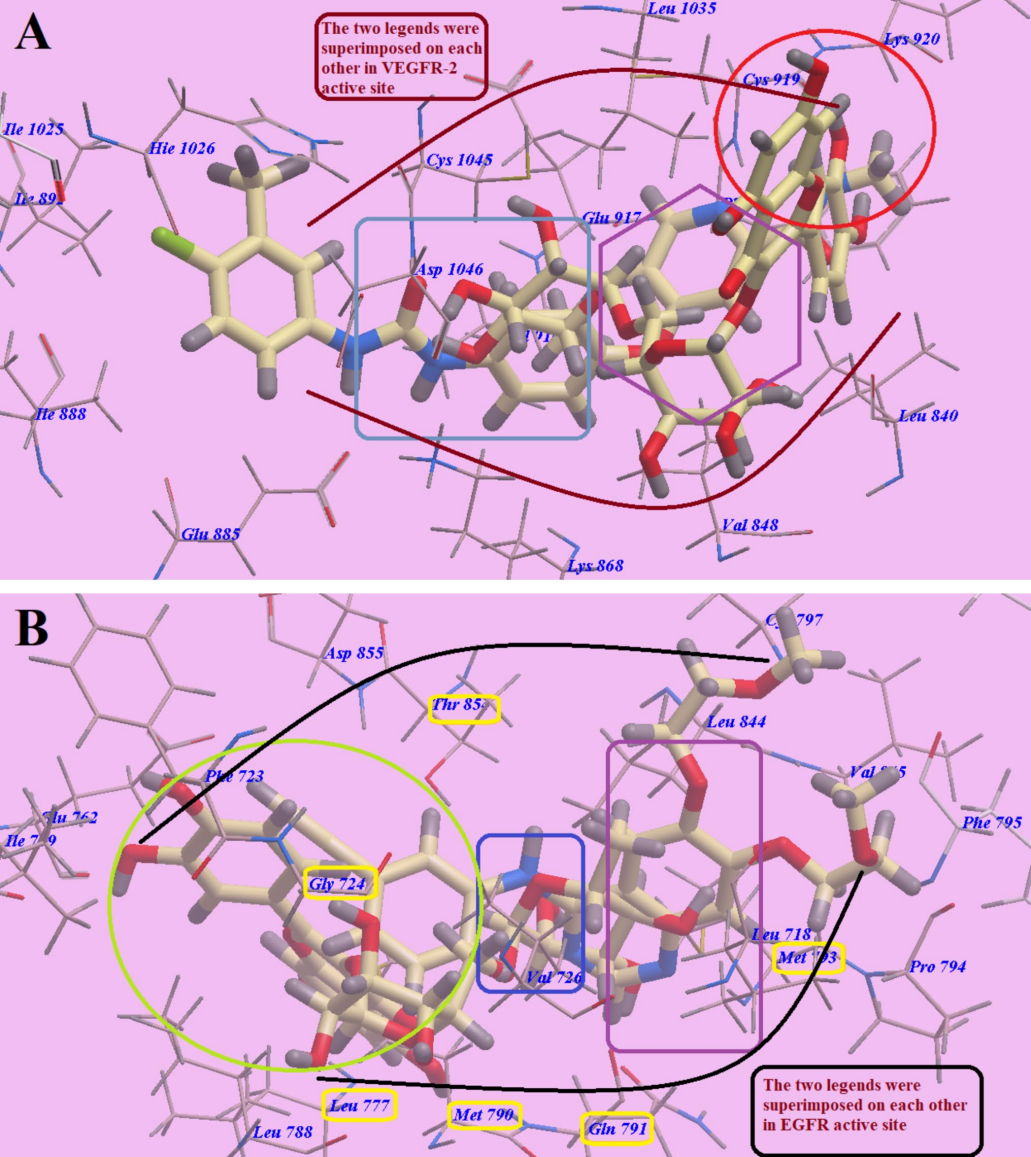
The superimposition of compound 4 with sorafenib (**A**) and erlotinib (**B**)

The aim of our study is to investigate the potential of a pectin/poly(acrylic acid) nanogel for the pH-responsive delivery of rutin as a dual VEGFR and EGFR inhibitor for cancer treatment. Rutin was employed for this study due to its pharmacophoric properties, which are essential for targeting both EGFR and VEGFR-2. Rutin binds in the same position and orientation as sorafenib in the VEGFR-2 binding site (Fig. 2A) and as erlotinib in the EGFR binding site (Fig. 2B), according to docking studies.

## Materials and methods

Pectin was purchased from Loba Chemie Indoaustranal Co. (Mumbai, India). The acrylic acid monomer (AAc) with 99% purity was purchased from Sigma-Aldrich Co. HCl (35.5 wt%), and sodium hydroxide were purchased from the local market.

### Mechanism of polymerization in nanogels induced by gamma irradiation nanogel

Crosslinking induced by radiation is a common technique to synthesize nanogels from water-soluble polymers like pectin and poly(acrylic acid). Here are some reasons why this approach works well (Matusiak et al. 2020). High-energy radiation like gamma rays can easily penetrate water easily and produce radical species that are reactive within the polymer chains. This process makes it possible to synthesize nanogels in mild environments, without requiring harsh chemicals or high temperatures (Ashfaq et al. 2021). The crosslinking of polymers induced by radiation is an approach to the synthesis of hydrogels and their nanoscale analogs, nanogels (NGs) (Dispenza et al. 2017). The research conducted by Rosiak et al. provides the optimal intramolecular crosslinking technique for the synthesis of nanogels (Rosiak et al. 2005). The process of radical formation in the chains of polymers initiates a series of intricate reactions that culminate in the formation of a crosslinked nanogel network. This phenomenon arises as a result of the interaction between the polymer chains and radiation, leading to the generation of free radicals along the polymer backbone. These free radicals play a pivotal role in intramolecular recombination reactions, where adjacent polymer chains interact and form new covalent bonds. This process, known as crosslinking, results in the creation of a three-dimensional network framework within polymers matrix. Crosslinking degree, or the extent to which these new bonds are formed, profoundly influences the properties and behavior of the resulting nanogel. Regarding drug delivery systems, crosslinked nanogel structure assumes paramount significance. The nanogel’s ability to encapsulate and subsequently release drug molecules hinges on the characteristics of this network. The degree of crosslinking governs important attributes such as the nanogel’s stability, swelling behavior, porosity, and responsiveness to external stimuli. A higher degree of crosslinking generally imparts greater structural stability and mechanical integrity to the nanogel. This can enhance its capacity to encapsulate therapeutic molecules, ensuring their retention within the nanogel’s matrix until specific triggers prompt their release. Conversely, nanogels with less crosslinking may be more responsive to external factors, potentially leading to a faster drug release profile. The crosslinked nanogel’s porosity and swelling behavior are also significantly influenced by the extent of crosslinking. A well-crosslinked nanogel might possess controlled swelling properties, allowing it to encapsulate a precise amount of drug molecules and regulate their release. Conversely, a less crosslinked nanogel could exhibit greater swelling, potentially accommodating a larger drug payload. In essence, the intricate interplay between radiation-induced radical formation, intramolecular recombination reactions, and the resulting crosslinked nanogel network underpins the foundation of a versatile and customizable drug delivery platform. By modulating the degree of crosslinking, researchers can fine-tune the nanogel’s properties to match specific therapeutic requirements. This ability to tailor the nanogel’s performance provides great potential for precise and controlled drug delivery, opening up avenues for more effective and efficient treatment strategies (Ulański et al. 1998).

In this study, the synthesis of Pec/PAAc nanogel was performed using an intramolecular crosslinking method facilitated by gamma irradiation. To prepare the solution, 0.05 g of pectin was dissolved in 10 ml of distilled water at room temperature (20–25 °C), yielding a 0.5% (w/v) pectin solution. Subsequently, 1 ml of acrylic acid (AAc) was added to this solution, resulting in a final concentration of 10% (w/v) acrylic acid. The mixture was stirred thoroughly to ensure complete dissolution of all components.

Next, subject the Pec/PAAc solution to sonication for 5 min. This process promotes even dispersion of the components throughout the solution, resulting in a uniform mixture.

Adjust the pH of the solution to 1 by gradually adding a 35.5% hydrochloric acid (HCl) solution. Carefully monitor the pH level during this process and make incremental adjustments until the pH stabilizes at 1. An effective polymerization reaction requires precise pH adjustment.

Then, expose the pH-adjusted solution to gamma irradiation using a 60Co gamma ray source. Follow appropriate safety protocols and use designated facilities, such as the NCRRT (Nuclear Chemistry and Radiation Technology) in Cairo, Egypt. Apply a gamma irradiation dose of 5 kGy to initiate the radical polymerization reactions.

The monomers of acrylic acid undergo radical polymerization as a result of gamma irradiation, which causes intramolecular crosslinking between the polymer chains. Pec and PAAc combine to form nanogel particles by this process, which produces a three-dimensional network structure.

The outlined procedure highlights the fundamental steps needed to synthesize Pec/PAAc nanogel using gamma irradiation as the radical polymerization initiator. Ensuring adherence to safety protocols and that the proper facilities and equipment are used, especially during the gamma irradiation phase. The resulting nanogel structures possess the potential for various applications, notably in controlled drug delivery systems and other areas that benefit from customized nanomaterial characteristics.

### Encapsulation of rutin inside (pectin/poly(acrylic acid)) nanogel

The suspension that is produced when the nanogel is synthesized using gamma irradiation is anticipated to have an acidic pH of around 1. To adjust the pH to a neutral value of 6, the following steps should be taken:

1. pH adjustment with sodium hydroxide (NaOH): A concentrated solution of NaOH gradually added to the (Pec/PAAc) suspension while being stirred magnetically at 150 rpm. The gradual rise in pH caused by this carefully timed addition of NaOH enables the nanogel particles to swell and modify their shape in response to the pH change. This slow process helps the nanogel decomplex and reconfigure as the pH gets closer to 6, which makes the structure less complex and more open.
2. Ultrasonic treatment: Following pH adjustment, the resulting solution (pH = 6) undergoes ultrasonic treatment. This step involves placing the nanogel suspension in an ultrasonic bath set to 70 W. The ultrasonic treatment lasts for 15 min and is conducted at room temperature. This treatment likely aids in further homogenizing and stabilizing the nanogel solution.

With the prepared nanogel solution, the next steps involve the incorporation of Rutin:

1. Preparation of rutin: To prepare rutin, 40 mg of the compound is dissolved in 10 ml of the (Pec/PAAc) solution. To ensure that the rutin is well integrated into the nanogel, slowly stir the mixture for 10 min.
2. pH adjustment of the mixture: The resulting mixture is subjected to pH adjustment to achieve a pH value of 3 using a 35.5 wt% HCl solution. The pH adjustment is a critical step to tailor the environment for optimal interactions between the nanogel and the encapsulated rutin.

The outlined procedure encompasses several essential stages, from adjusting the pH of the nanogel suspension to incorporating rutin into the nanogel matrix. These steps collectively contribute to the controlled and systematic preparation of the (Pec/PAAc) nanogel and the incorporation of the therapeutic compound, rutin, for potential applications in drug delivery and related fields. It's imperative to execute these steps meticulously, maintaining precise conditions and adhering to safety protocols, to ensure the desired properties and functionality of the resulting nanogel-Rutin complex.

### Fourier transform infrared spectroscopy

FTIR spectra of the pure rutin and the Pec/PAAc/rutin nanogel were acquired in order to study the chemical stability of rutin within the nanogel. Each sample was compressed into discs and combined with 500 mg of potassium bromide powder that was IR-grade for this examination. The spectrum was obtained between 4000 and 600 cm^−1^.

### Particle size, zeta potential, and morphology

Particle size was measured at 25 °C by dynamic light scattering using a Malvern Zeta-sizer Nano-ZS (Malvern Instruments, UK). Prior to each measurement, the dispersions were diluted as necessary to obtain the ideal light scattering intensity. The same device was used to test the zeta potential by tracking the motion of particles in an electric field following a 1:100 dilution. Results are presented as the mean of three independent experiments, with standard deviation included (Al-Zuhairy et al. 2023).

A transmission electron microscope (TEM, JEM-1230, Joel, Tokyo, Japan) was used to analyze the morphology of the prepared nanogel. After being diluted, the nanogel was applied on a grid covered in carbon. After that, it was stained negatively using a 2% w/v solution of phosphotungstic acid. The material was stained, allowed to dry at room temperature, and then examined under a microscope. Sample was dried at room temperature before being observed under the microscope.

### Determination of drug content and spreadability

The chosen nanogel (equivalent to 1 mg of rutin) was weighed and dissolved in 20 mL of ethanol. The colloidal mixture was sonicated for 20 min to ensure complete dissolution of the rutin into the ethanol. Following sonication, the dispersions was filtered using 0.45-μm membrane filter, and the resultant filtrate was then diluted with additional ethanol. An aliquot of this dispersion was subjected to scanning at a wavelength of 356 nm using a UV spectrophotometer (Shimadzu UV1650 Spectrophotometer, Kyoto, Japan). The drug content was subsequently calculated in triplicates (Ali et al. 2022). Additionally, the spreadability of the rutin nanogel was assessed using a glass slide apparatus (El-Adl et al. 2024). One milligram of nanogel was enclosed between two glass slides. The amount of time it took for the top slide to separate from the stack of slides was noted. Equation (1) was then used to calculate the gel spreadability.

1 $$S= \frac{ML}{T}$$

where *S* is for spreadability, *M* is for weight attached to the higher slide (g), *L* is for glass slide length (cm), and *T* is for the amount of time the slide takes to separate from the stack of slides.

### Ex vivo skin permeation study

The study evaluated the drug diffusion potential of a prepared rutin nanogel formulation and a control rutin suspension using a vertical Franz diffusion cell apparatus. Hairless rat dorsal skin was frozen, stabilized with pH 7.4 phosphate buffer, and mounted in the Franz cell (Hassan et al. 2022). The receptor compartment was filled with pH 7.4 phosphate buffer, maintained at 32 ± 0.5 °C with constant stirring. The nanogel formulation and control were then applied to the excised skin. Samples were collected from the acceptor compartment and analyzed spectrophotometrically at 356 nm. This data was used to determine the drug flux at steady state (Jss), the permeability coefficient (Kp), and the enhancement ratio. The Jss was calculated by plotting the cumulative drug penetration per unit area (μg/cm^2^) versus time and finding the slope of the linear portion. The Kp was then calculated by dividing the Jss by the initial drug concentration (C_0_) (Abdou et al. 2017).

### Short-term storage study

Maintaining the integrity of formulations over time requires assessing their chemical and physical stability under varied storage conditions. The formulation was kept for 90 days at 4 °C and 25 °C in sealed glass vials to assess the physical stability of rutin nanogel. For analysis, samples were obtained from each vial at 0-, 45-, and 90-day intervals. For analysis, samples were extracted from each vial at 0-, 45-, and 90-day intervals. Particle size, zeta potential, and drug content were assessed as previously mentioned at the end of the storage time. These values were compared to those of the fresh developed nanogel using a Student’s *t* test, with a significance level set at *p* ≤ 0.05. Moreover, the formulation was visually examined for any indications of physical changes, drug leakage, or aggregation (Shoman et al. 2023a).

### Docking studies

To examine its binding interactions with the enzymes VEGFR-2 and EGFR^T790M^, rutin was subjected to docking studies. The Molsoft program, which offers specific capabilities for simulating protein–ligand interactions, was used to carry out the docking simulations (El-Hddad et al. 2024). This software expects the binding of tiny, flexible molecules (such as substrates or potential drugs) to proteins with established 3D structures, by using grid interaction potentials (http://www.molsoft.com/icm_pro.html).

This research employed VEGFR-2 (PDB ID 4ASD) and EGFR^T790M^ (PDB ID 3W2O) as biological targets (Elmetwally et al. 2019). Their 3D structures were sourced from the Brookhaven Protein Data Bank (https://www.rcsb.org/). Specifically, sorafenib and erlotinib, which are known inhibitors of VEGFR-2 and EGFR^T790M^, were used as reference ligands to assess the accuracy of the docking predictions in comparison to experimental data.

The protein was prepared for docking by addition of polar hydrogen to the protein atoms. The protein binding site was defined by placing a grid over the center of co-crystallized ligand. Before a protein was ready for docking simulations, all the necessary grid maps were calculated prior to docking. Our compound was drawn as a 3D structure, and its energy was minimized. The ligand was extracted from the binding site, and the compound discussed herein was docked into the binding site.

### In vitro cytotoxic activity

Numerous cancer cell lines were procured from the American Type Culture Collection (ATCC) situated in Manassas, USA. These lines included hepatocellular carcinoma (HepG2), colorectal carcinoma (HCT-116), and breast cancer (MCF-7). In particular, the Roswell Park Memorial Institute (RPMI 1640) medium was used to cultivate these cancer cells according to the proper protocol. The culture medium was supplemented with penicillin (100 units/mL), streptomycin (100 mg/mL), and heat-inactivated fetal bovine serum (10%). The cultivation of these cancer cells was carried out in a humidified atmosphere with 5% (v/v) CO_2_ at a temperature of 37 °C.

The 3-[4,5-dimethylthiazole-2-yl]-2,5-diphenyltetrazolium bromide (MTT) assay was used to evaluate the cytotoxicity of different substances or treatments. A commonly used approach for assessing the viability and proliferation of cells, including the cancer cell lines described, is colorimetric analysis.

Actively proliferating cells from different cancer cell lines were first trypsinized and quantified. They were then seeded into 96-well microtiter plates at appropriate densities, ranging from 2000 to 10,000 cells per 0.33 cm^2^ well. These seeded cells were incubated in a humidified environment at 37 °C for 24 h to allow for cell attachment and acclimation.

Following the initial incubation, the cells were subjected to different doses of the chemicals under investigation, ranging from 0.1 to 1000 μM, for a duration of 72 h. The MTT assay was used to evaluate the treated cells’ vitality. Specifically, the culture media were removed, and the cells were incubated with 200 μl of 5% MTT solution per well.

Due to this, the MTT dye was metabolized by the living cells over the course of 2 h, forming colorful, insoluble formazan crystals.

This allowed the viable cells to metabolize the MTT dye into colored, insoluble formazan crystals over a 2-h period. After this incubation, the formazan crystals were dissolved in 200 μl of acidified isopropanol per well for 30 min after the residual MTT solution was withdrawn. This dissolution step was carried out at room temperature with a MaxQ 2000 plate shaker, covered with aluminum foil and continuously shaking.

The absorbance of the dissolved formazan crystals was measured at 570 nm through the use of Stat FaxR 4200 plate reader. Viability of the cells was calculated as a percentage of the untreated control, and Graph Pad Prism version 5 software was used to calculate the concentration at which the compounds triggered 50% of the maximal inhibition of cell growth (IC_50_).

### In vitro VEGFR-2 kinase assay

Analyzing the assay of VEGFR-2 kinase involved utilizing the Alpha Screen system (PerkinElmer, USA) and an anti-phosphotyrosine antibody in accordance with the manufacturer’s protocol. A solution containing 50 mM Tris–HCl (pH 7.5), 5 mM MnCl2, 5 mM MgCl2, 0.01% Tween-20, and 2 mM DTT was used to conduct the enzyme reactions. A total of 10 μM ATP, 0.1 μg/mL of biotinylated poly-GluTyr (in a 4:1 ratio), and 0.1 nM of VEGFR-2 (Millipore, UK) were all added in the reaction mixture.

Prior to initiating the catalytic reaction with ATP, the investigated substances were subjected to a 5-min room temperature incubation period with the VEGFR-2 enzyme, with final concentrations varying between 0 and 300 μg/mL.

In order to terminate the reactions, 25 μL of a solution comprising 100 mM EDTA, 10-μg/mL donor and acceptor streptavidin beads were added to 62.5 mM HEPES (pH 7.4), 250 mM NaCl, and 0.1% BSA. After an overnight dark incubation, the plate was read using an ELISA Reader (PerkinElmer, USA). The baseline control was represented by wells with biotinylated poly-GluTyr (4:1) and the enzyme but without ATP, and the reaction control was provided by wells with the substrate and the enzyme but not the tested compounds.

The percentage inhibition was computed by comparing contrasting the compound-treated samples with the control incubations. The concentration-inhibition response curve (based on triplicate measurements) was used to identify the concentration of the test drug that induced 50% inhibition (IC_50_), and the results were compared with sorafenib (Sigma-Aldrich, USA), which acted as the standard VEGFR-2 inhibitor.

### In vitro assay for inhibiting EGFR^T790M^ kinase activity

The study utilized the homogeneous time resolved fluorescence (HTRF) assay to evaluate the kinase activity of EGFR^T790M^ mutant. The EGFR^T790M^ enzyme and ATP were obtained from Sigma. Initially, the tested chemical was first incubated for 5 min with the EGFR^T790M^ enzyme and its substrates in an enzymatic buffer. This pre-incubation step allowed the compound to interact with the enzyme before the catalytic reaction was initiated. Afterwards, 1.65 μM of ATP was added to the reaction mixture to initiate the enzymatic process. The assay was carried out at room temperature for 30 min. The samples were mixed with detection reagents containing EDTA to stop the enzymatic process. The detection phase was allowed to proceed for 1 h. By utilizing GraphPad Prism 5.0 software, the inhibitory potency of the tested compounds was determined by calculating the half-maximal inhibitory concentration (IC_50_) values. Three independent measurements were done to determine the concentration of each compound.

### In silico ADMET analysis

The pkCSM online tool was utilized to forecast the pharmacokinetic and toxicity characteristics of the examined substances (https://biosig.lab.uq.edu.au/pkcsm/prediction). The compounds were first prepared by hand-drawing and energy minimization according to the standard small molecule protocol. Then, using the pkCSM prediction algorithm, the ADMET (absorption, distribution, metabolism, excretion, and toxicity) descriptors were determined. This comprehensive in silico assessment provided insights into the pharmacokinetic and toxicological characteristics of the tested substrates.

## Results and discussion

### Mechanism of polymerization in nanogels induced by gamma irradiation nanogel

The use of gamma irradiation is a versatile method for the synthesis of nanogels, which are intricate networks of crosslinked polymer chains. With this method, gamma radiation is applied to the polymer chains, causing the production of free radicals within them. As a result, hydrogel nanoparticles with precisely controlled and customizable properties are often produced using this approach.

**Fig. 3:**
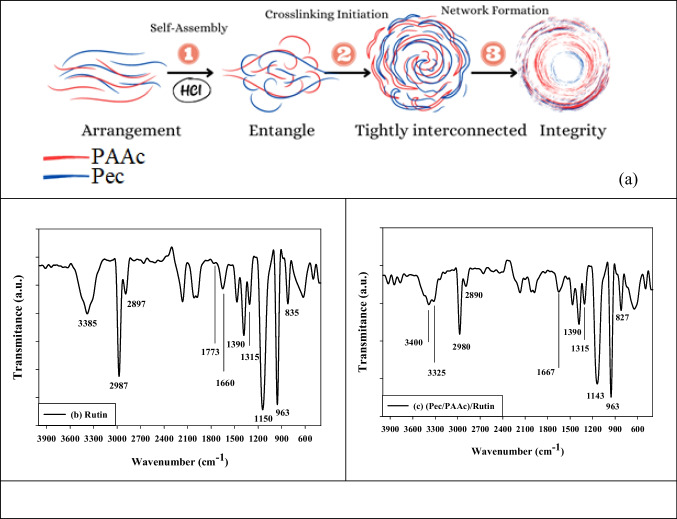
The suggested mechanism for nanogel synthesis via gamma irradiation (**a**), the FTIR spectrum of rutin (**b**), and the FTIR spectrum of (Pec/PAAc)/rutin (**c**)

As illustrated in Fig. 3a, the process by which gamma irradiation forms nanogels includes carefully choosing monomers and polymers that can undergo radiation-induced crosslinking reactions. The required monomers are added to a polymer solution or dispersion, where the polymers maintain desirable properties including hydrophilicity and biocompatibility that are useful for drug loading in biomedical applications.

Formation of nanogels in this specific case involves the utilization of poly(acrylic acid) (PAA) and pectin as the polymeric components. Hydrogen bonds are created between the two kinds of polymers as a result of interactions between the carboxylate groups (-COO-) on the PAA chains and the pectin molecules. The hydroxyl groups (-OH) on both the PAA and pectin further engage in hydrogen bonding interactions in acidic (low pH) environments. This interplay between the functional groups serves to bridge the PAA and pectin chains, leading to the creation of a loosely crosslinked network. This initial step is referred to as self-assembly, where the attractive forces between the functional groups of polymer chains cause the chains to start interacting and entwining.

The second phase begins with the application of gamma irradiation as a crosslinking agent. In this process, water subjected to radiolysis, which results in the production of reactive species such as hydroxyl radicals (•OH) and hydrated electrons (e-). These radicals interact with the polymer chains, causing hydrogen abstraction. The resulting interactions promote the creation of crosslinks, which are covalent bonds that link neighboring polymer chains. As a result, a densely interconnected three-dimensional network structure is created (Shoman et al. 2023b).

As nanogel formation process progresses, the crosslinks continue to form, resulting in increasingly tighter interconnections among the polymer chains. This progression culminates in the creation of a comprehensive three-dimensional network structure. This network effectively entraps the solvent within, providing nanogels their distinctive gel-like consistency. Over time, the creation of the nanogel achieves an equilibrium state in which the rate of network disintegration or rearrangement equals the rate of crosslinking. These crosslinks maintain the integrity of the nanogel structure by stabilizing it.

In summary, gamma irradiation–induced nanogel production is a multi-step process. It begins with the initial self-assembly of the polymers through hydrogen bonding, followed by the introduction of crosslinking agents that generate reactive radicals. These reactive species then promote the formation of covalent crosslinks, leading to the creation of a tightly interconnected three-dimensional network. This intricate process ultimately results in the establishment of a stabilized nanogel structure. The ability to tailor the properties of nanogels through this gamma irradiation-based synthesis method makes them versatile candidates for diverse applications, particularly in the realm of controlled drug delivery and biomedical interventions.

### FTIR analysis of nanogel formation via gamma irradiation

Fourier transform infrared spectroscopy (FTIR) is a versatile analytical method employed to recognize and investigate the functional groups within a system. It provides information about the molecular structure and chemical composition of a compound by measuring the interactions between different types of bonds and infrared light. In the case of rutin, which is a flavonoid compound found in various plants, FTIR can reveal the vibrational modes and characteristic peaks associated with its functional groups. Figure 3b provided FTIR for rutin corresponding to specific vibrational modes of functional groups within the molecule. The peak at 3385 cm^−1^ is likely associated with the O–H stretching vibration of the multiple hydroxyl (-OH) groups present in Rutin, particularly in the sugar moieties and phenolic rings. The peaks at 2987 cm^−1^ and 2897 cm^−1^ correspond to the asymmetric and symmetric C-H stretching vibrations in the aliphatic groups, possibly from the carbohydrate portion of the rutin structure.

The characteristic peak at 1773 cm^−1^ is associated with the carbonyl (C = O) stretching vibration, often attributed to the lactone ring in rutin. The peak at 1660 cm^−1^ suggests the presence of C = C stretching vibrations, commonly found in aromatic rings. The peak at 1390 cm^−1^ corresponds to the bending vibrations of C-H bonds in aliphatic groups, while the peak at 1315 cm^−1^ might indicate the presence of C-O stretching vibrations, possibly arising from ether or ester functional groups. The peak at 1150 cm^−1^ could relate to C-O stretching vibrations, which might be attributed to the glycosidic linkages in rutin’s sugar moieties. The peak at 963 cm^−1^ may indicate the presence of aromatic C-H bending vibrations, likely associated with the phenolic rings in rutin. The peak at 835 cm^−1^ is consistent with the presence of aromatic C-H bending vibrations, further reinforcing the presence of phenolic rings. The positions and intensities of these FTIR peaks provide valuable information about the interaction between (Pec/PAAc) and rutin molecules, as well as the composition and functional groups present in their compound.

The FTIR spectrum of the (Pec/PAAc)/rutin nanogel, as shown in Fig. 3c, reveals several characteristic peaks that provide insights into the interactions between the nanogel components and the Rutin molecule. The peaks at 3400 cm^−1^ and 3325 cm^−1^ correspond to the O–H stretching vibrations, indicating the presence of hydroxyl groups contributed by both the (pectin/PAAc) nanogel and the rutin compound, respectively. The peak at 2980 cm^−1^ suggests the presence of asymmetric C-H stretching vibrations, likely originating from the aliphatic components of the pectin and poly(acrylic acid) in the nanogel. Similarly, the peak at 2890 cm^−1^ is associated with the symmetric C-H stretching vibrations in aliphatic groups. The broad peak at 1667 cm^−1^ indicates the presence of C = O stretching vibrations, which may be attributed to the various carbonyl-containing functional groups present in the (Pec/PAAc)/rutin nanogel. The peak at 1390 cm^−1^ might represent the C-H bending vibrations in the aliphatic structures of the nanogel components. The peaks at 1315 cm^−1^ and 1143 cm^−1^ relate to the C-O stretching vibrations, likely associated with the glycosidic linkages or other oxygen-containing groups in the nanogel and rutin. The peak at 963 cm^−1^ indicates the presence of aromatic C-H bending vibrations, suggesting the incorporation of the phenolic rings from the rutin compound into the nanogel structure. Finally, the peak at 827 cm^−1^ may correspond to the C-H bond bending vibrations in aliphatic groups.

By comparing the observed peaks in Fig. 3c with the reference FTIR spectrum of rutin in Fig. 3b, and the known functional group vibrations, the changes in peak positions, intensities, and shapes provide strong evidence of the interactions between the (Pec/PAAc) nanogel and the rutin molecule.

### PH-dependent release of rutin from (pectin/poly(acrylic acid)) nanogels

**Fig. 4:**
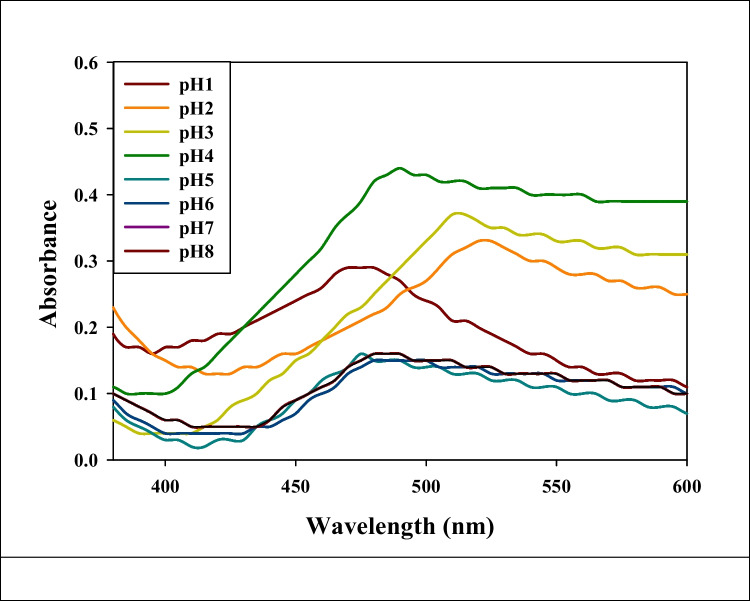
UV visible spectroscopy analysis of rutin released from the (Pec/PAAc) nanogel

**Fig. 5:**
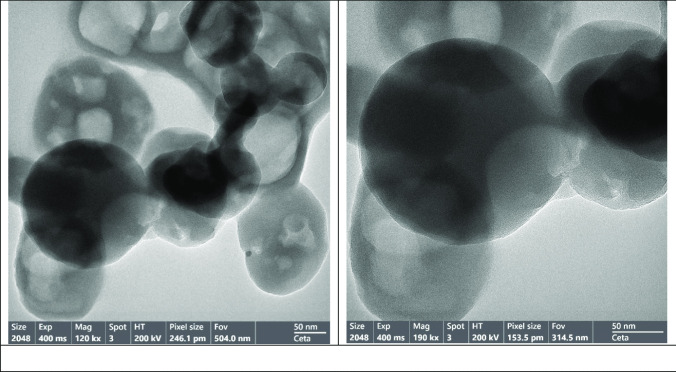
Surface morphology of (Pec/PAAc) rutin nanogel system

The pH-responsive behavior observed in Fig. 4 highlights a compelling avenue for advancing targeted drug delivery systems. This intriguing characteristic of the nanogel/rutin complex indicates its potential to precisely control rutin release based on environmental pH variations. This pH-sensitive release mechanism considers great promise for therapeutic applications. It offers a pathway to significantly improve the accuracy of drug delivery systems. By harnessing this pH-responsive property, the nanogel/rutin complex could usher in a new era of drug delivery precision. The ability to modulate drug release based on pH levels opens doors to targeted delivery strategies tailored to specific sites or tissues within the body. This is particularly advantageous in instances where localized pH variations occur, such as in the acidic environment of the stomach or in disease microenvironments. The complex’s aptitude for releasing its therapeutic payload selectively at the targeted site could significantly boost the effectiveness of treatment. A paramount advantage arising from this pH-sensitive release system is the potential reduction in undesirable side effects. Through the fine-tuned control of drug release, the risk of adverse reactions impacting non-target tissues could be minimized. This facet of targeted drug delivery has the potential to elevate patient comfort and compliance during the course of treatment, further enhancing overall therapeutic outcomes. Optimization of drug dosage is a pivotal aspect of effective treatment, and the pH-responsive behavior of the nanogel/rutin complex offers a path to achieving this optimization. The controlled manner in which the therapeutic agent is released enables a more precise and regulated dosing schedule. As a result, patients may experience improved adherence to treatment plans, potentially leading to enhanced therapeutic success rates. Importantly, the pH-triggered release mechanism could also play a pivotal role in mitigating toxicity concerns associated with certain drugs. By directing drug release primarily to the target site, healthy tissues might be spared exposure to potentially toxic agents. This reduction in toxicity risk could offer a significant advancement in patient safety and overall treatment outcomes. The noteworthy increase in rutin release observed from pH 1 to pH 4 about 65% underscores the dynamic behavior of the nanogel complex. This intriguing phenomenon likely arises from the pH-dependent swelling or degradation of the nanogel matrix, ultimately causing the release of encapsulated rutin. This understanding paves the way for tailored modifications to the nanogel composition, enabling fine-tuning of the pH range for optimal drug release. In conclusion, the pH-responsive behavior demonstrated by the nanogel/rutin complex, as depicted in Fig. 5, holds substantial promise for revolutionizing targeted drug delivery systems. The ability to modulate drug release to pH fluctuations offers a suite of advantages, ranging from targeted delivery and enhanced efficacy to reduced side effects and minimized toxicity. Continued research and development in this realm could yield innovative drug delivery platforms with far-reaching implications for therapeutic interventions.

In the pH range of 1 to 3, an interesting behavior is observed with the nanogel/rutin complex. During this acidic pH range, the nanogel maintains its structural integrity and exhibits minimal rutin release. This phenomenon can be attributed to the interaction dynamics within the complex. At lower pH levels, specifically from 1 to 3, the carboxyl groups present on poly(acrylic acid) chains become protonated. This protonation alters the charge distribution along the PAAc chains, leading to a non-ionic interaction scenario. Importantly, this change in charge state contributes to the stability of the nanogel structure. An essential factor in this stability is the intermolecular hydrogen bonding interactions that occur between the pectin (Pec) and poly(acrylic acid) (PAAc) molecules. These interactions, facilitated by hydrogen bonds, are essential to maintain the structural cohesion of the nanogel complex. The formation of hydrogen bonds between Pec and PAAc reinforces the complex’s overall architecture, preventing premature or excessive rutin release. In essence, the absence of rutin release at acidic pH levels is a result of the concerted interplay between the protonation of carboxyl groups on PAAc chains, the ensuing non-ionic interactions, and the stabilizing hydrogen bonding between Pec and PAAc molecules. This intricate balance of molecular interactions ensures that the nanogel remains intact and retains its cargo under these specific pH conditions, holding promise for controlled and targeted drug delivery applications.

At the specific pH value of 4, an intriguing event known as “pH-triggered release” occurs within the nanogel/rutin complex. This event is marked by a decomplexation process between the two primary polymer components, namely pectin (Pec) and poly(acrylic acid) (pAAc), which together constitute the matrix of the nanogel.

The decomplexation process is driven by the pH-responsive behavior of the pAAc chains. As the pH rises to 4, the PAAc chains begin to release protons, converting carboxyl groups into negatively charged carboxylate ions. This shift in charge distribution induces repulsive forces between the negatively charged pAAc chains and the corresponding charged segments within the Pec chains.

The electrostatic repulsive forces between the pAAc and Pec chains trigger a sequence of events within the nanogel structure. As repulsive forces gain prominence, the nanogel network undergoes a process of swelling and partial breakdown. This phenomenon causes the nanogel structure to open up, forming pores or gaps within its matrix. The consequences of this structural transformation are significant. The openings or pores generated in the nanogel matrix allow the trapped rutin molecules, which were previously encapsulated within the complex, to diffuse out. This diffusion process is now facilitated by the newly created pathways within the nanogel. Consequently, the increase in rutin release observed at pH 4 can be attributed to this “pH-triggered release” mechanism. The decomplexation-induced nanogel swelling and structural alteration result in the exposure of previously encapsulated rutin molecules to the surrounding environment. This liberation enables rutin to diffuse freely through the opened nanogel structure, leading to an enhanced release of its constituent compounds.

In the alkaline pH range spanning from pH 5 to pH 8, an interesting phenomenon emerges, characterized by a significant reduction of approximately 66% in the release of rutin from the nanogel complex. This behavior can be attributed to the influence of (Na) counter ions, which exhibit a noticeable increase in concentration as the pH shifts towards the higher alkaline values.

At these elevated pH levels, the presence of (Na) counter ions give rise to a phenomenon known as the “charge screening effect”. This effect entails the neutralization or shielding of charged functional groups within the nanogel structure (Ghobashy and Bassioni 2018). The screening of charges can facilitate the reformation of the nanogel network, reducing its porosity and consequently restricting the rutin release. Specifically, the negatively charged carboxylate groups on the pAAc chains, which are crucial components of the nanogel, become subject to shielding due to the interaction with the (Na) counter ions.

The shielding effect initiated by the (Na) counter ions introduces a layer of electrostatic repulsion between the carboxylate groups, which were previously instrumental in the pH-triggered release mechanism, and the surrounding (Na) ions. This repulsion attenuates the interactions that had previously facilitated the controlled release of rutin. As a consequence, the structural dynamics of the nanogel complex are altered. The shielding effect reduces the porosity of the nanogel matrix, hindering the diffusion and release of rutin molecules from within. This restricted diffusion limits the extent to which rutin can permeate through the nanogel structure, thus leading to the observed decrease in rutin release.

### Morphology of (pectin/poly(acrylic acid)) nanogel

A transmission electron microscope (TEM) image of the (pectin, PAAc) nanogel encapsulating rutin offers a detailed visualization of the nanogel’s morphology and structure on a micro- to nanoscale level. TEM, which utilizes electron beams for scanning, delivers high-resolution images that reveal the surface features, textures, and overall structure of the nanogel. The image shows the morphology of the nanogel as a collection of spherical or irregularly shaped nanoparticles formed from the crosslinked pectin and PAAc chains. Detailed observations of the surface texture may reveal features such as bumps, pores, or ridges, providing insights into the polymer interactions and the encapsulation of rutin. The TEM image also allows for the assessment of the size distribution of the nanogel particles, including average particle size and variability. Additionally, regions within the nanogel particles where rutin is encapsulated might be discernible, appearing as darker or lighter spots. The image may also reveal aggregation or clustering of nanogel particles, potentially due to electrostatic interactions, hydrogen bonding, or other forces.

### Evaluation the effect of pH on the size and zeta potential of (Pec/PAAc) rutin nanogel

The pH of a solution is a critical parameter that can have a profound impact on the behavior of molecules and complexes within it. In the realm of nanogel/drug complexes, the adjustment of pH from highly acidic (pH 1) to moderately alkaline (pH 8) has been explored in a fascinating study, shedding light on how pH influences the size and stability of these complexes.

One of the most notable findings of this study is the direct relationship between pH and the size of (Pec/PAAc)/Ru nanogel/drug complexes. As the pH of the solution increases, so does the particle size of the complexes. This observation provides valuable insights into the optimization of these complexes for various applications, particularly in drug delivery and nanomedicine.

At pH 1, the complexes exhibit a relatively large particle size, which is expected due to the acidic conditions. However, as the pH gradually increased, a striking trend emerges. The smallest particle size, a mere 16 nm, is achieved at pH 3. This represents a crucial discovery because it indicates that pH 3 is the point at which the nanogel/drug complexes attain their most stable and compact form. At this pH, the nanogel structure appears to be tightly bound to the drug molecule, resulting in a minimal hydrodynamic radius and, consequently, a diminutive particle size.

A particularly intriguing phenomenon is the sharp increase in particle size between pH 4 and 7. Within this range, there is a noticeable transition in the behavior of the complexes. The particle size grows from 185 nm at pH 4 to 296 nm when the pH is adjusted to 7. This abrupt and substantial size change suggests that there exists a critical pH window within this range. During this window, the nanogel structure undergoes significant structural changes, leading to increased swelling. The exact mechanisms behind these changes warrant further investigation, but it is likely related to the deprotonation of specific groups within the nanogel structure, causing the conformation to become less compact. Consequently, the hydrodynamic radius increases, resulting in larger particle sizes.

As the pH surpasses 7 and moves into moderately alkaline conditions, an intriguing phenomenon occurs. The particle sizes start to level off, indicating a pH threshold, presumably above 7, beyond which further structural changes in the nanogel have limited impact on size. This leveling-off effect suggests that at higher pH levels, the nanogel complexes reach a stable configuration where additional structural adjustments yield diminishing returns.

Conversely, when the pH drops below 3, well into the acidic range, the nanogel/drug complexes exhibit larger particle sizes. This can be attributed to the protonation of specific groups within the nanogel structure, leading to a more compact conformation. It is a clear demonstration of how pH, through protonation and deprotonation processes, can dramatically influence the size and stability of these complexes.

The implications of these findings are far-reaching, especially with regard to drug delivery and biomedical applications. The capability for precisely controlling the size and stability of nanogel/drug complexes by adjusting pH offers a valuable tool for tailoring drug release kinetics and optimizing therapeutic efficacy. Future research in this field may delve deeper into the molecular mechanisms underpinning pH-induced changes in nanogel structure and explore how these alterations affect drug release profiles. Additionally, investigating the correlation between pH and the zeta potential of the complexes can provide further insights into their stability and electrostatic interactions.

In conclusion, this study underscores the significance of pH as a critical factor in the behavior of (Pec/PAAc)/Ru nanogel/drug complexes. pH directly influences their size and stability, with pH 3 being the optimal condition for achieving small, stable complexes. The sharp size increase between pH 4 and 7 suggests a critical pH range where substantial structural transformations occur. The ability to harness these pH-dependent properties holds immense promise for advancing drug delivery and biomedical applications, offering opportunities for more precise and effective therapies.

**Fig. 6:**
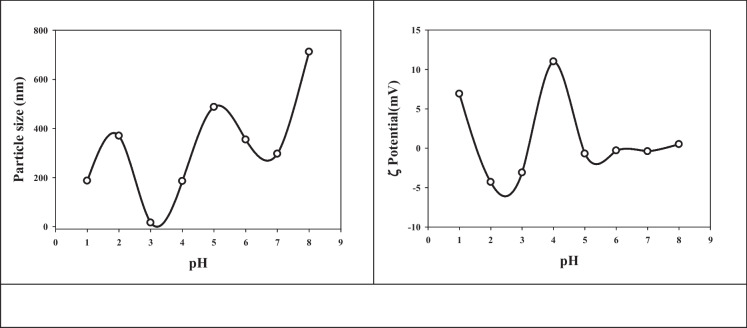
Dummy pH dependent of **a** particle size distribution (nm) and **b** zeta potential of (Pec/PAAc)/rutin samples

Figure 6b illustrates that the zeta potentials of the (Pec/PAAc)/Ru samples exhibit considerable variation as the pH is adjusted from 1 to 8. Notably, the zeta potential values do not seem to correlate directly with the particle sizes, indicating that the surface charge is largely independent of particle size variations induced by pH changes. The zeta potential values reveal an intriguing pattern. At pH 1, pH 4, and pH 8, the nanogel particles carry positive zeta potentials, with values around 6.96, 11, and 0.5 mV, respectively. Conversely, at pH 2 and pH 6, the zeta potentials are slightly negative, with pH 2 showing the most negative charge (− 4.3 mV) and pH 6 showing the least negative charge (− 0.3 mV).

One striking observation is that the zeta potential values do not exhibit a discernible trend in relation to pH. Unlike the particle size, which exhibited a systematic increase with increasing pH, the zeta potential values fluctuate within a range of 11 to − 0.3 mV across the pH range of 1 to 8. This suggests that there may not be a clear correlation between pH variations and the surface charge characteristics of the nanogel particles. One possible explanation for the complex surface charge behavior observed in the (Pec/PAAc) rutin nanogel complexes is the dissociation of acrylic acid (AAc) molecules inside the nanogel matrix. AAc is known to be a weak acid, capable of both donating and accepting protons (H + ions) depending on the pH of the surrounding medium. This behavior leads to fluctuations in the surface charge as pH varies, as seen in the experimental results. Understanding the zeta potential behavior of these nanogel/drug complexes is of paramount importance in various applications, particularly in drug delivery and stability assessments. The surface charge can influence interactions with biological systems and other particles in suspension. The observation of both positive and negative zeta potentials across the tested pH range highlights the complexity of these systems. Future research could delve deeper into the molecular mechanisms responsible for the observed zeta potential variations. Additionally, exploring the consequences of these zeta potential changes on the stability, colloidal behavior, and drug release kinetics of the nanogel/drug complexes could provide valuable insights into optimizing their performance for specific biomedical applications. In conclusion, the zeta potential values of (Pec/PAAc)/Ru nanogel/drug complexes exhibit a diverse and intriguing pattern across the pH range of 1 to 8. These surface charge variations are largely independent of changes in particle size induced by pH adjustments. The complex behavior of zeta potential, influenced by the dissociation of AAc molecules within the nanogel structure, underscores the need for a comprehensive understanding of these systems to harness their potential in drug delivery and other biomedical applications.

### Determination of drug content and spreadability

The cumulative drug content from the nanogel was found to be 86.14 ± 2.61%. This result indicates that the drug was uniformly dispersed throughout the nanogel formulation. This uniformity can be attributed to the effective swelling of the polymeric system at pH 3, which facilitated the entrapment of the drug within the nanogel matrix. Concurrently, spreadability is a vital attribute when assessing nanogels for use in topical use. The chosen nanogel exhibited a spreadability of 55.26 ± 2.67 g·cm/s by weight, indicating good ease of application and optimal consistency for effective spreading on the skin.

### Ex vivo skin permeation study

**Fig. 7:**
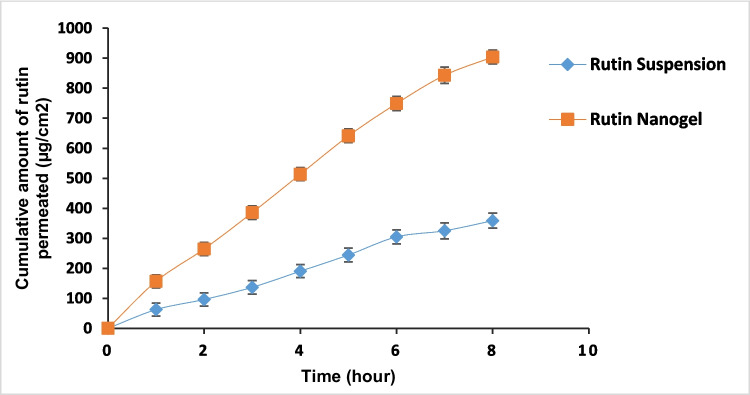
Ex vivo permeation profiles of rutin from prepared nanogel and rutin suspension (control) (*n* = 3)

Hairless dorsal skin was used in ex vivo permeation investigations to evaluate drug diffusion and compare it with rutin suspension. The findings, shown in Fig. 7, demonstrated that the drug suspension had a lower permeability coefficient (0.019 cm/h) and a smaller cumulative amount permeated over 8 h (358.705 μg) compared to the nanogel formulation (0.046 cm/h and 903.829 μg), yielding an enhancement ratio of 2.389. The marked increase in transdermal permeation indicated that incorporating rutin into a nanogel significantly improved its permeability and drug diffusion. This improvement is likely due to the nanogel’s smaller particle size, which increases surface area, its mucoadhesive properties that prolong skin retention, its ability to enable controlled release, and its enhanced stability (Marwah et al. 2016; Ay Şenyiğit et al. 2021).

### Short-term storage study

After being stored for 45 and 90 days at 4 °C and 25 °C, the rutin nanogel formulation showed no visible signs of aggregation or any alteration in appearance. The findings presented in Table 1 exhibit the particle size (PS) zeta potential, drug content, and spreadability for both the freshly prepared and stored formulations, revealing no significant variations (*p* > 0.05 for all parameters). Furthermore, there were no noticeable changes in the physical characteristics of the nanogel, such as color.

**Table 1:** The impact of storage parameters on the characterization of the formulated rutin nanogel was examined at 45 and 90 days, and at 4 °C and 25 ± 3 °C. Data are shown as mean ± SD (*n* = 3)

Parameters	Fresh nanogel	Rutin nanogel after 45 days at 4 °C	Rutin nanogel after 45 days at 25 °C	Rutin nanogel after 90 days at 4 °C	Rutin nanogel after 90 days at 25 °C
Particle size (nm)	185.0 ± 5.1	184.2 ± 4.7	184.9 ± 6.1	185.3 ± 6.5	185.4 ± 7.2
Zeta potential (mV)	11.00 ± 0.07	11.06 ± 0.03	11.01 ± 0.02	11.05 ± 0.08	11.08 ± 0.01
Drug content	86.14 ± 2.61	85.16 ± 3.61	86.34 ± 2.61	85.14 ± 1.61	85.14 ± 1.61
Spreadability	55.26 ± 2.67	54.68 ± 1.36	56.75 ± 2.45	55.36 ± 1.24	56.46 ± 2.98

### Docking studies

Molecular docking studies were conducted using Molsoft software. The experiments employed VEGFR-2 and EGFR^T790M^ with PDB IDs 4ASD (Khedr et al. 2021; Aziz et al. 2022) and 3W2O (Gandin et al. 2015), respectively.

#### VEGFR-2 inhibitors

**Fig. 8:**
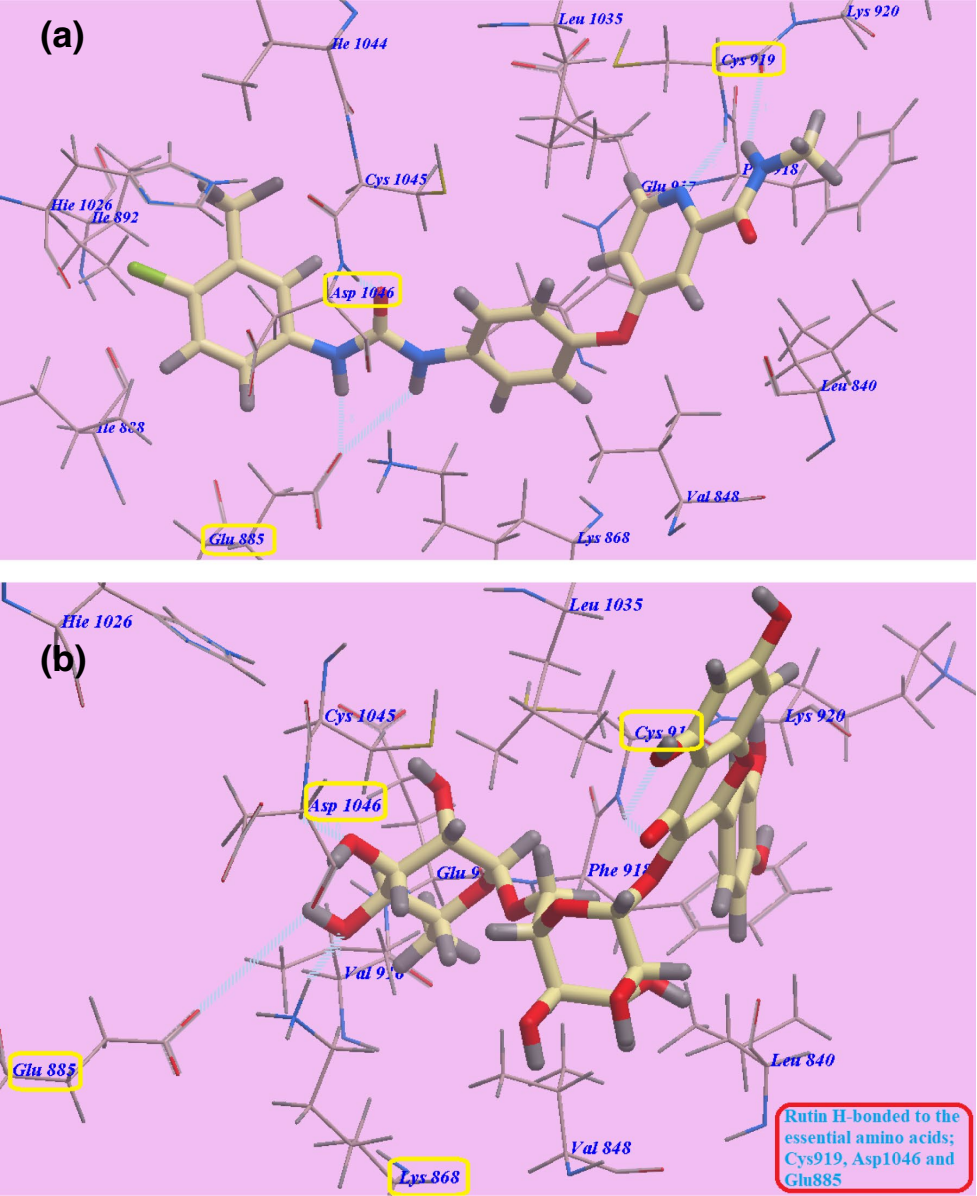
Predicted binding configurations: **a** sorafenib with 4ASD and **b** rutin with 4ASD, highlighting hydrogen bonds with dotted lines

The molecular docking analysis revealed the recommended binding mode for sorafenib, which generated five hydrogen bonding connections and showed an affinity value of − 99.50 kcal/mol. Specifically, sorafenib established two hydrogen bonds with *Glutamate885* (1.77 Å and 2.75 Å), one hydrogen bond with *Aspartate1046* (1.50 Å), and two hydrogen bonding interactions with *Cysteine919* (2.51 Å and 2.10 Å) within the VEGFR-2 active site (Fig. 8a).

The suggested binding configuration of rutin virtually resembles that of sorafenib, exhibiting an even higher affinity value of − 111.05 kcal/mol and forming five hydrogen bonds. Rutin established two hydrogen bonds with *Cysteine919* (2.95 Å and 2.98 Å) in the ATP-binding site, and it also extended its interactions over the gate area to engage with the essential amino acids *Aspartate1046* (1.58 Å) and *Glutamate885* (2.96 Å). Additionally, rutin formed an extra hydrogen bond with *Lysine868* (2.97 Å) (Fig. 8b). Based on these observations, rutin was classified as a Type II VEGFR-2 inhibitor, similar to the mode of action of sorafenib. The comparative analysis of the binding modes and affinities of sorafenib and rutin suggests that rutin has the potential to be a potent VEGFR-2 inhibitor, exhibiting a higher binding affinity and a comparable binding mode to the approved drug sorafenib.

#### EGFR^T790M^ inhibitors

**Fig. 9:**
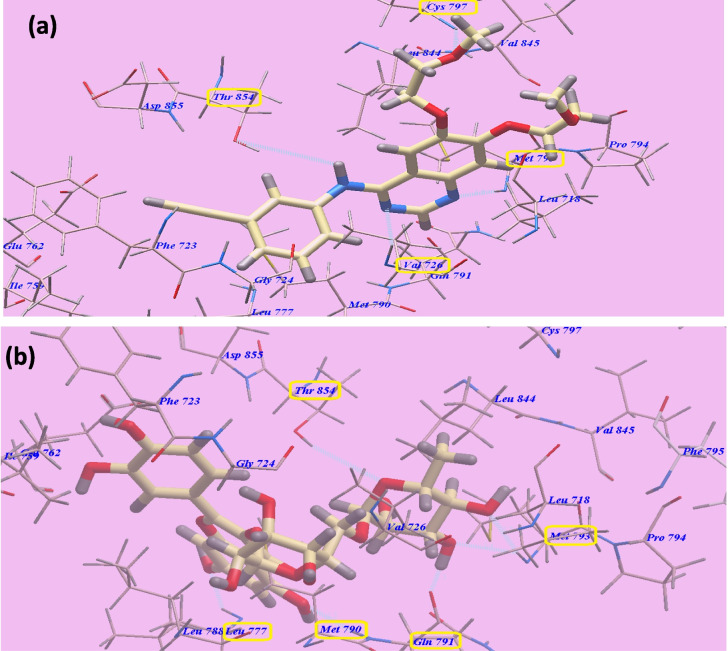
Predicted binding configurations of **a** erlotinib with 3W2O and **b** rutin with 3W2O, showing hydrogen bonds

The molecular docking analysis also revealed the binding mechanism of erlotinib into the ATP-binding region of the VEGFR-2 kinase domain. The anticipated binding mode for erlotinib displayed a binding energy of − 82.77 kcal/mol and formed four hydrogen bonds. Erlotinib formed two hydrogen connections with *Valine726* (2.97 Å) and *Methionine793* (1.82 Å) through its quinazoline ring. *Cysteine797* (2.05 Å) and one of the two 2-methoxyethoxy groups bonded through a hydrogen bond. The NH spacer generated a hydrogen bond with the essential amino acid *Threonine854* (2.99 Å). *Phenylalanine723*, *Glycine724*, *Valine726*, *Isoleucine759*, *Glutamate762*, *Leucine777*, *Methionine790*, *Glutamine791*, *Threonine854*, and *Aspartate855* create hydrophobic region I, which is occupied by the 3-ethynylphenyl head of erlotinib. Moreover, *Leucine718*, *Methionine793*, *Proline794*, *Leucine844*, and *Valine845* defined hydrophobic area II, which was occupied by the 2-methoxyethoxy tail of erlotinib (Fig. 9a).

The suggested binding mechanism of rutin within the ATP-binding region of the VEGFR-2 kinase domain is further shown by the molecular docking research to be remarkably resembles that of erlotinib.

Rutin formed six hydrogen bonds with key amino acid residues in the VEGFR-2 ATP-binding site, demonstrating an affinity value of − 111.44 kcal/mol. Specifically, rutin established three hydrogen bonds with the crucial amino acids *Methionine793* (at distances of 2.37 Å and 2.72 Å) and *Threonine854* (at a distance of 2.98 Å). Additionally, rutin formed three hydrogen bonds with *Leucine777* (at a distance of 1.75 Å), *Methionine790* (at a distance of 2.35 Å), and *Glutamine791* (at a distance of 2.95 Å) (Fig. 9b).

The strong binding affinity of rutin, along with the formation of multiple hydrogen bonding interactions with key residues within the VEGFR-2 ATP-binding site, suggests that rutin could potentially exhibit potent inhibitory activity against the VEGFR-2 kinase domain. The observed binding mode of rutin, which is similar to that of the known VEGFR-2 inhibitor erlotinib, provides further evidence for the potential of rutin as a promising anti-angiogenic therapeutic agent targeting the VEGFR-2 signaling pathway.

#### In vitro cytotoxicity and inhibition assays for VEGFR-2 and EGFR^T790M^ kinases

Four human cancer cell lines were used to evaluate the cytotoxic activity of rutin (**3**) and its nanogel formulation (**4**): hepatocellular carcinoma (HepG2), lung cancer (A549), breast cancer (MCF-7), and colorectal carcinoma (HCT-116). The assessment was performed using the standard MTT colorimetric assay, as per Mosmann’s description (Freimoser et al. 1999; Mosmann 1983). In order to establish a comparative baseline, the experiments also included the reference cytotoxic drugs sorafenib and erlotinib. The cytotoxic activity results are summarized in Table 2, which presents the growth inhibitory concentration (IC_50_) values of the tested compounds. The results indicate that the anticancer effects against each of the four tested cancer cell lines were markedly enhanced by the nanogel formulation. Specifically, the nanogel formulation reduced the IC_50_ values against HepG2, A549, MCF-7, and HCT-116 cells by 58.19%, 81.29%, 71.81%, and 67.16%, respectively, compared to the free rutin (**3**).

**Table 2:** In vitro cytotoxicity and kinase inhibition of the prepared compounds

Compounds	IC_50_ (μM)^a^					
	HepG2	A549	MCF-7	HCT116	VEGFR-2	EGFR^T790M^
Rutin (3)	14.88	8.71	12.31	15.59	1.18	0.66
Rutin nanogel (4)	6.22	1.63	3.47	5.12	0.83	0.21
Sorafenib	4.00	4.04	5.58	5.05	0.84	^b^NT
Erlotinib	7.73	5.49	8.20	13.91	^b^NT	0.24

^a^IC_50_ values are mean of three separate experiments

^b^*NT* Compounds not tested

Furthermore, the rutin compound (**3**) and its nanogel formulation (**4**) were evaluated for their inhibitory effects on VEGFR-2 and mutant EGFR^T790M^ kinases. the homogeneous time resolved fluorescence (HTRF) assay (Jia et al. 2006) for EGFR^T790M^ and an anti-phosphotyrosine antibody with the Alpha Screen system (Abou-Seri et al. 2016) for VEGFR-2 were used in the present investigation.

The results revealed that both the rutin compound (**3**) and its nanogel formulation (**4**) exhibited stronger inhibitory effects against VEGFR-2 and EGFR^T790M^ kinases compared to the free rutin (**3**). Specifically, the nanogel formulation (**4**) reduced the IC_50_ values for VEGFR-2 and EGFR^T790M^ by 29.66% and 68.18%, respectively, compared to the free rutin (**3**) (Table 2).

These outcomes show that the nanogel formulation (**4**) was more effective in inhibiting the VEGFR-2 and EGFR^T790M^ kinases than the free rutin compound (**3**), which suggests improved targeting and potency of the nanogel-based delivery system.

In the literature, pectin-based nanomaterials are one strategy of delivering drugs to the colon known as colon-targeted drug delivery system (DDS). Pectin’s swelling properties, as well as its capacity to withstand gastrointestinal degradation, have made it a popular carrier for colon-specific medication delivery. The biodegradability and gel-forming nature of this polysaccharide are the characteristics that drive its selection as a carrier for specific medication delivery (Karlsen et al. 2021). In this study, we used pectin-based nanogel as DDS carrier for rutin for treatment of four different cancer cell lines HepG2, A549, MCF-7, and HCT-116.

#### In silico ADMET analysis

An in silico analysis was done to assess the physicochemical characteristics and predicted ADMET profile of rutin **(3**). The methods of the pkCSM descriptor algorithm were used to do this analysis (Pires et al. 2015) and compared against the guidelines outlined in Lipinski’s rule of five (Lipinski et al. 2012). According to the rule of five, a molecule has a fair chance of having strong absorption capabilities if it satisfies three out of the following criteria: a molecular weight of less than 500, a partition coefficient (logP) of no more than 5, a maximum of five hydrogen bond donors, and a maximum of ten hydrogen bond acceptors are the requirements. The analysis revealed that the reference drug sorafenib violated one of these rules, while the rutin nanogel formulation (**4**) violated three rules. In contrast, the reference drug erlotinib did not fail to meet any of Lipinski’s five criteria.

The in silico analysis revealed that the nanogel formulation (**4**) exhibits a low predicted gastrointestinal absorption in humans, with a value of 17.81 (Table 3). This suggests that compound (**4)** may be more suitable for administration through routes other than the oral/gastrointestinal tract (Beig et al. 2013). Regarding the ability of compound to penetrate the CNS, the analysis showed that the synthesized compound (**4**) possesses the capability to access the CNS. The predicted CNS permeability value for compound 4 is − 5.726, which is lower than the values for sorafenib (− 2.007) and erlotinib (− 3.216). The lower CNS permeability of compound (**4)** compared to sorafenib and erlotinib indicates that the synthesized compound is expected to have a reduced likelihood of causing CNS-related side effects. This is a potentially favorable characteristic, as it may provide an improved safety profile compared to the standard anticancer agents.

**Table 3:** ADMET profile of rutin (compound 4), sorafenib, and erlotinib

Parameters	Rutin	Sorafenib	Erlotinib
Physical and chemical attributes			
Molecular weight	610.521	464.831	393.443
Log P	− 1.6871	5.5497	3.4051
Rotatable bonds	6	5	10
Acceptors	16	4	7
Donors	10	3	1
Surface area	240.901	185.111	169.532
Absorption			
Water solubility	− 2.952	− 4.822	− 4.736
Caco2 permeability (Papp)	− 0.561	0.689	1.431
Human intestinal absorption	17.81	89.043	94.58
Skin permeation	− 2.735	− 2.767	− 2.741
P-glycoprotein substrate	Confirmed	Confirmed	Not confirmed
Inhibition of P-glycoprotein-I	Not detected	Detected	Detected
Inhibition of glycoprotein-II	Not detected	Detected	Detected
Distribution			
Human VDss	− 2.735	− 0.29	0.199
Human unbound fraction	0.227	0.065	0.059
Permeability throughout BBB	− 2.725	− 1.684	− 0.745
CNS permeability	− 5.726	− 2.007	− 3.216
Metabolism			
CYP2D6 Substrate	Not confirmed	Not confirmed	Not confirmed
CYP3A4 Substrate	Not confirmed	Confirmed	Confirmed
Inhibition of CYP3A4	Not detected	Detected	Detected
Inhibition of CYP2D6	Not detected	Detected	Detected
Inhibition of CYP2C9	Not detected	Detected	Detected
Inhibition of CYP2C19	Not detected	Not detected	Not detected
Inhibition of CYP1A2	Not detected	Detected	Detected
Excretion			
Clearance	− 0.019	− 0.219	0.702
Renal OCT-2 substrate	Confirmed	Not confirmed	
	Not confirmed		
Toxicity			
AMES toxicity	Not detected	Not detected	Not detected
Maximum tolerated dose in humans	0.643	0.549	0.839
Inhibition of hERG-I	Not detected	Not detected	Not detected
Inhibition of hERG-II	Detected	Detected	Detected
Acute toxicity (LD_50_)	2.471	2.538	2.393
Chronic toxicity (LOAEL)	4.881	1.198	1.37
Hepatic toxicity	Not detected	Detected	Detected
Skin sensitization	Not detected	Not detected	Not detected
Toxicity to *Tetrahymena pyriformis*	0.285	0.383	0.309
Minnow toxicity	7.345	0.189	− 0.1

The in silico ADMET analysis revealed significant distinctions between the synthesized compound 4 and the reference drugs sorafenib and erlotinib. While sorafenib and erlotinib are known to inhibit the major drug-metabolizing enzyme CYP3A4, compound (**4**) did not exhibit this inhibitory effect, likely due to the higher lipophilicity of the reference drugs. Additionally, erlotinib showed a higher clearance rate compared to compound **4** and sorafenib, suggesting it would be eliminated more quickly and require shorter dosing intervals. In contrast, slower clearance rate of compound (**4**) implies a longer duration of action. Importantly, compound (**4**) did not share the hepatotoxic effects observed with sorafenib and erlotinib. Furthermore, compound (**4**) demonstrated larger oral chronic toxic levels, suggesting a possible increase in safety margin. Overall, the analysis suggests that compound (**4**) has a more favorable ADMET profile compared to the reference drugs.

## Conclusion

In the landscape of drug delivery systems, the emergence of pectin/polyacrylic acid nanogel as versatile carriers holds immense promise for advancing therapeutic interventions. This study explored the synthesis, characterization, and potential applications of these nanogels, focusing on their pH-responsive behavior and encapsulation of rutin. The synthesis of nanogel via polymerization using gamma irradiation showcases a powerful approach that leverages radiation-initiated crosslinking of polymers, resulting in a three-dimensional network with tunable properties. The demonstrated pH-responsive behavior of the nanogel complex opens new avenues for precise drug release strategies, particularly in environments characterized by distinct pH levels. The intriguing pH-triggered release mechanism observed at pH 4, driven by polymer deprotonation and structural shifts, holds significant potential for targeted drug delivery. Additionally, the impact of charge screening in the alkaline pH range further expands the applications of these nanogel. The encapsulation of rutin within the nanogel matrix exemplifies the versatility of this platform, with a systematic procedure involving pH adjustment, ultrasonic treatment, and controlled introduction of rutin optimizing drug interactions and release profiles. Moreover, FTIR analysis enhances our understanding of molecular interactions within the nanogel-rutin complex, providing insights into the fundamental forces at play. Notably, the rutin nanogel demonstrated significantly enhanced drug permeation through rat skin compared to the drug suspension, underscoring its superior ability to penetrate biological barriers.

Furthermore, the nanogel was identified as a dual inhibitor targeting both VEGFR-2 and EGFRT790M, forming critical hydrogen bonds with key residues, classifying it as a type II VEGFR-2 inhibitor. Our compound demonstrated improved anticancer efficacy across four cancer cell lines, achieving significant reductions in IC50 values for HepG2, A549, MCF-7, and HCT-116 by 58.19%, 81.29%, 71.81%, and 67.16%, respectively. Additionally, it decreased IC50 values for VEGFR-2 and EGFRT790M by 29.66% and 68.18%, respectively. In silico analyses revealed a favorable ADMET profile for the compound, characterized by fewer central nervous system side effects, and no evidence of liver toxicity, with chronic toxic dosages greater than reference drugs. These attributes highlight the potential of pectin/polyacrylic acid nanogel as a foundation for developing more effective and targeted dual inhibitors of VEGFR-2 and EGFRT790M, thereby enhancing anticancer treatment options. This approach provides a strong foundation for further exploration and research.

## Data Availability

All source data for this work (or generated in this study) are available upon reasonable request.
